# Recto-Femoral Fistula Presenting as Emphysematous Cellulitis of the Knee: A Case Report & Literature Review

**DOI:** 10.1155/2011/479209

**Published:** 2011-09-08

**Authors:** P. K. Karampinas, D. S. Evangelopoulos, I. S. Benetos, E. Kavroudakis, J. Vlamis

**Affiliations:** Third Department of Orthopaedics, University of Athens Medical School, Athens, Greece

## Abstract

*Purpose*. The rectofemoral fistula represents a devastating complication of colorectal surgery. Its early diagnosis and treatment are critical to obtain a good patient outcome. *Case Presentation*. A 75-year-old Caucasian female patient presented with high fever, ileus, low back pain, sciatic nerve palsy, and infection of the right knee. After numerous surgical debridements and antibiotic therapies, a rectofemoral fistula was diagnosed. *Conclusion*. Increased doctors' alertness is mandatory for the early identification and surgical treatment of patients suffering from a rectofemoral fistula before the stage of diffuse infection has significantly decreases their postoperative morbidity and mortality.

## 1. Introduction

Colon diseases may lead to devastating complications. One of the most challenging complications to diagnose and treat for the orthopaedic surgeon is a recto-femoral fistula [1, 2]. Diagnosis can be difficult due to the variety of clinical symptoms, including low back pain with sciatic nerve palsy, femoral thrombophlebitis, anaerobic soft tissue infection, emphysematous cellulitis, and gas gangrene of the lower limb [3–6]. 

Pus drainage and extensive debridement of all necrotic tissues along with the reconstruction of the colon are considered as the most appropriate and effective treatment. However, the results of surgical treatment depend mostly on its prompt recognition, the judgement, and the surgical skill of the surgeon [3, 5, 7]. Due to the increased postoperative morbidity and mortality, such patients require intense postoperative medical and nursing care [3, 8].

## 2. Case Presentation

A 75-year-old Caucasian female presented to the Internal Medicine Department with high fever, diffuse abdominal pain, and pseudodiarrhea. After thorough clinical and laboratory testing, ileus was diagnosed and the patient was admitted to the department. She did not recall any history of malaise, fatigue, night sweating, weight loss, or preceding trauma. Her past medical history revealed a left hip hemiarthroplasty, 5 years ago, due to a subcapital femoral fracture. 

**Figure 1:**
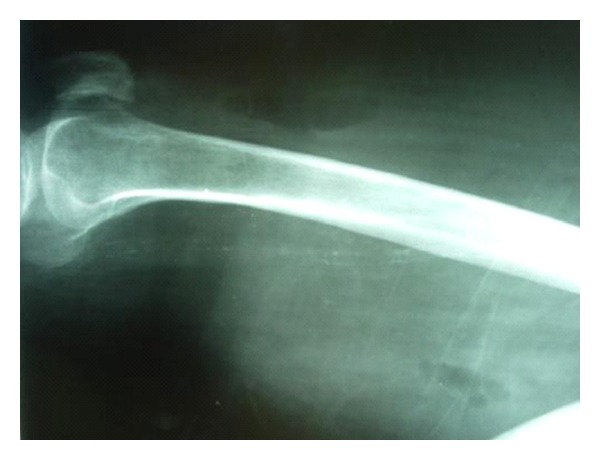
Plain lateral radiograph shows the right femur. Subcutaneous emphysema is observed as gas in the fascial planes between muscles and subcutaneous tissues.

**Figure 2:**
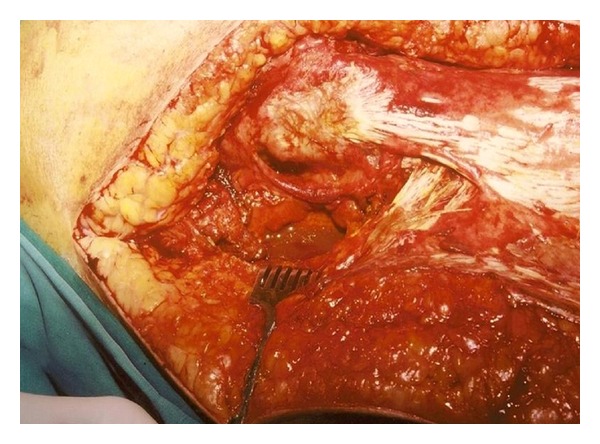
The surgical wound at the hip region. Pus, fecal material, and air bubbles can be observed coming out of the great sciatic foramen after the patient was asked to cough.

**Figure 3:**
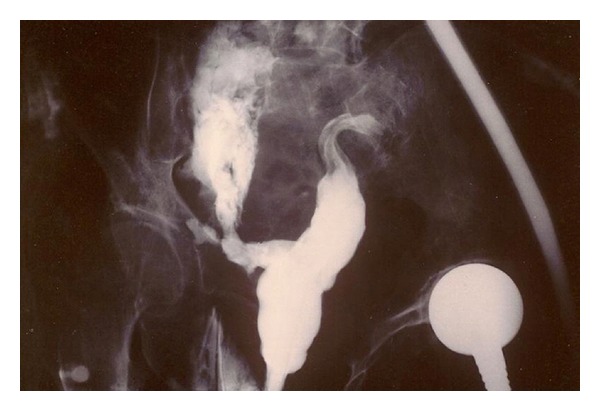
Plain anteroposterior radiograph showing the pelvis 5 minutes after per rectum administration of gastrografin. A rectal perforation allows gastrografin to escape into the pelvic cavity.

**Figure 4:**
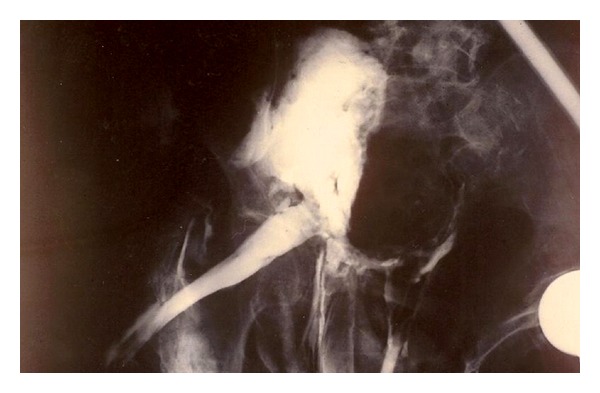
Plain anteroposterior radiograph showing the pelvis 7 minutes after per rectum administration of gastrografin. Gastrografin is escaping from the pelvic cavity through a fistula to the thigh.

On admission, she had fever (38.2°C) attributed partially to a urinary tract infection as well as to the infected knee joint. *Pseudomonas aeruginosa* was isolated from urine cultures. After administration of appropriate antibiotic therapy, the patient's body temperature returned to normal. However, despite the initial treatment of the ileus, consisting of enemas, correction of fluid and electrolyte imbalance, and administration of appropriate antibiotics and analgesics, the patient continued to experience diffuse abdominal pain, flatulence, and pseudodiarrhea. Lumbar pain and sciatica were added to the clinical picture. The clinical examination of the right lower limb revealed a positive Lasègue sign at 45° as well as reduced muscle power, sensation, and tendon reflexes. At this stage, cauda equina syndrome was considered to be the most probable diagnosis. However, few days later, the patient became feverish again (38.5°C) and had dyspnea and profuse watery diarrhea. The patient's general condition started to rapidly deteriorate. Blood cultures revealed an anaerobic organism, and parenteral nutrition was administered along with a triple scheme of antibiotic therapy (teicoplanin, netromycin, and ciprofloxacin). Two weeks later, patient's right knee joint became reddish, hot, and edematous while palpation revealed crepitation. Emphysematous cellulitis of the knee was diagnosed at this stage. Surgical debridement was immediately performed, revealing a seropurulent wound discharge. *E. coli* and *Enterococcus* were isolated from the obtained cultures. Despite the thorough debridement and the appropriate adjustment of the antibiotic therapy (penicillin, vancomycin, and amikacin), the patient remained febrile and the surgical wound purulent. A new wound culture revealed *Pseudomonas aeruginosa* and *E. coli*, while a blood culture revealed a gram-negative microorganism. The patient's general condition continued to deteriorate with dyspnea, tachypnea, cyanosis, Hippocratic face, and decreased level of consciousness. Radiologic examination of the right femur demonstrated the presence of gas between the muscles and subcutaneous tissue (Figure 1). Within 24 hours from the last deterioration, under epidural anesthesia, the anterolateral surface of the right thigh was dissected. Approximately 2 liters of pus were drained from the space between the muscles and the totally detached fascia, and thorough mechanical fluid lavage and tissue debridement were performed. Purulent fluid resembling diarrhea along side with gas bubbles was observed while the surrounding tissues were necrotic (Figure 2). Since fecal discharge and gas bubbles were seen coming out of the greater sciatic foramen, during provoked coughing, a fistula between the intestine and the wound was suspected to be the cause of the patient's condition. A general surgeon performed a rectal digital examination and recognised a rectal perforation 5 cm proximal to the anal ring. Radiologic examination of the pelvis by means of a gastrografin enema revealed the presence of a recto-femoral fistula treated surgically with an emergency sigmoidostomy (Figures 3 and 4). During the next 19 days, mechanical lavages were routinely performed and appropriate antibiotic treatment was administered. On the 20th postoperative day, the distal end of the surgical wound was closed.

## 3. Discussion

Several colon diseases may result in the formation of internal or external fistulae. The most frequent causes include Crohn's disease and colon diverticulitis; however, carcinomas of the colon, foreign bodies, and rectal trauma have been implicated as less frequent causes [1]. In addition, there have been numerous reports of fistulae involving pelvic structures following pelvic radiotherapy [8]. 

While the majority of the external fistulae involve the anterior abdominal wall, internal fistulae may involve any pelvic structure. The most common sites of extra-abdominal perforation are the buttocks and the hips. Although perforation to the lower extremities is less common, perianal and hip fistulae have been reported [9, 10]. 

Diagnosis of a recto-femoral fistula can be challenging due to the variety of clinical symptoms. The detection of bacteria (usually more than one species) commonly found in the gastrointestinal tract in the wound culture mandates proper examination of the colon to exclude a connection between the colon and the infected area. 

Pus drainage and extensive debridement of all necrotic tissues along with the reconstruction of the colon are considered to be the most appropriate and effective surgical management of an internal fistula to the lower limb. However, many patients die due to progressive deterioration of their general condition despite appropriate surgical management [1, 2]. The purpose of presenting this case report was to increase every clinical doctor's alertness to this condition. This may lead to early identification and surgical treatment of patients suffering from a recto-femoral fistula before the stage of diffuse infection has begun which significantly decreases their postoperative morbidity and mortality.

## 4. Conclusion

A fistula between the colon and the knee joint is extremely rare, serious, and potentially fatal. Prompt recognition of such conditions by means of triple contrast computed tomography and appropriate surgical management can significantly decrease postoperative morbidity and mortality. 

##  Consent

Written informed consent was obtained from the patient's next of kin for publication of this case report and accompanying images. A copy of the written consent is available for review by the Editor-in-Chief of this journal.

##  Authors' Contribution

All authors certify that they have participated sufficiently in the intellectual content the analysis of data. Each author has reviewed the final version of the manuscript and approves it for publication. Should the editors request the data upon which the work is based, the authors shall produce it.
